# Effects of Freezing, Frozen Storage and Thawing on the Water Status, Quality, Nutrition and Digestibility of Meat: A Review

**DOI:** 10.1002/fsn3.70774

**Published:** 2025-08-07

**Authors:** Yingying Zhu, Jiaying Zhu, Xuebin Shi, Mingdong Fan

**Affiliations:** ^1^ Food & Medicine Homology Big Health Innovation Consortium, Engineering Research Center of Magnetic Resonance Analysis Technology, Department of Food Nutrition and Test Suzhou Polytechnic University Suzhou Jiangsu China; ^2^ Key Laboratory of Meat Processing and Quality Control, College of Food Science and Technology Nanjing Agricultural University Nanjing Jiangsu China

**Keywords:** freeze, frozen meat, meat nutrition, meat quality, thaw

## Abstract

Meat is highly perishable and susceptible to microbial spoilage and quality deterioration. Freezing is one of the most widely used and effective ways for preserving meat. However, freezing, frozen storage, and thawing can lead to changes that affect meat quality. The formation and subsequent melting of ice crystals can damage muscle cells and oxidize muscle components such as protein and fat, resulting in meat quality deterioration and loss of nutritional value. This review summarizes recent studies on the effects of these processes on the water status, quality, nutrition, and digestibility of meat, which explains how the formation, growth, and recrystallization of ice crystals cause water migration, cell damage, protein oxidation, lipid oxidation, water holding capacity reduction, and nutrition degradation. The aim is to provide theoretical guidance and research directions for controlling quality degradation, developing freezing and thawing technologies, and improving the quality and nutritional value of frozen meat.

## Graphical abstract

Freezing induced ice crystal formation and recrystallization damage muscle cells, promoting protein/lipid oxidation and water loss, thereby degrading meat quality and nutrition. Optimized freezing–thawing methods (e.g., rapid freezing, novel thawing, and advanced packaging) mitigate structural damage and oxidative stress. Future innovations in preservation technologies are critical for enhancing frozen meat quality and digestibility.

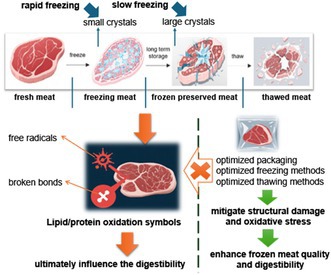

Meat provides essential nutrients for humans, including proteins, fat, amino acids, vitamins, and minerals (Geiker et al. 2021; Pourya et al. 2021; Stadnik 2024). However, due to its high susceptibility to spoilage, preservation techniques are required to ensure safety and extend shelf life (Saucier 2016; Zhou et al. 2010). Freezing is one of the most widely used and effective methods for preserving meat quality (Köprüalan Aydın et al. 2023). It is utilized as the primary method for national meat reserves, market regulation, and large‐scale industry storage. The global market for frozen muscle foods is projected to reach $100 billion by 2028 (Xie et al. 2023).

Freezing is characterized as a physical process that involves the formation of ice crystals within the moisture content of meat (Ali et al. 2015). This initiates water migration, leading to an increase in solute concentration in unfrozen cell fluid and the creation of osmotic pressure gradients (Egelandsdal et al. 2019). Water shifts towards areas of lower osmotic pressure, resulting in cell dehydration and the concentration of intracellular fluid. This disrupts the cellular environment, lowers muscle pH, and compromises the stability of pigment and enzymatic proteins (Pan et al. 2020; Zhang et al. 2017; Ashour et al. 2020). Additionally, calcium ion concentration is elevated by freezing, activating calcium‐dependent proteases that accelerate the degradation of contractile proteins (Peng et al. 2021; Wang, Liang, et al. 2020; Wei et al. 2020). Furthermore, key antioxidant enzymes are inactivated, thereby promoting oxidative reactions such as lipid oxidation and protein denaturation (Li, Zhu, and Sun 2018; Zhang 2019).

Thawing is required for frozen meat. During thawing, the ice crystals in the meat melt into liquid water, which is subsequently reabsorbed by the cells, theoretically returning the meat to its pre‐frozen state (Li et al. 2019; Pan et al. 2020; Köprüalan Aydın et al. 2023). However, during meat freezing and storage, the formation, growth, and recrystallization of ice crystals initiate a cycle of water transfer both inside and outside the cells (Xie et al. 2023). The extracellular water is solidified into ice crystals at low temperatures (Zhu et al. 2023). As temperatures continue to decrease, these ice crystals expand, absorbing surrounding water and causing the cell membrane to rupture, ultimately damaging the tissue structure (Zhang and Ertbjerg 2019; Ali et al. 2015). When the temperature rises during thawing, the ice crystals melt into water, but the muscle tissue has already been damaged by the ice crystals; the water status and distribution in the meat are significantly different compared to fresh meat (Shao et al. 2018; Qian et al. 2022; Mulot et al. 2019). The repeated transitions between water and ice result in varying degrees of structural damage and denaturation of key meat components, such as protein, fat, and vitamins (Zhang et al. 2023; Cheng, Wang, et al. 2019; Ma et al. 2015). Consequently, these changes lead to a deterioration in meat quality attributes, including water holding capacity, color, texture, flavor, gelation, and emulsification properties, ultimately affecting its edibility and suitability for further processing (Zhang et al. 2023).

This review summarizes recent studies on the effects of freezing, frozen storage, and thawing processes on the water status, quality, nutrition, and digestibility of meat, to provide theoretical guidance and research direction for controlling the quality degradation, developing freezing and thawing technology, and enhancing the quality and nutritional characteristics of frozen meat, which is of great significance for promoting the development of the frozen meat industry.

## 1 Effects of Freezing, Frozen Storage and Thawing on the Water Status of Meat

**FIGURE 1:**
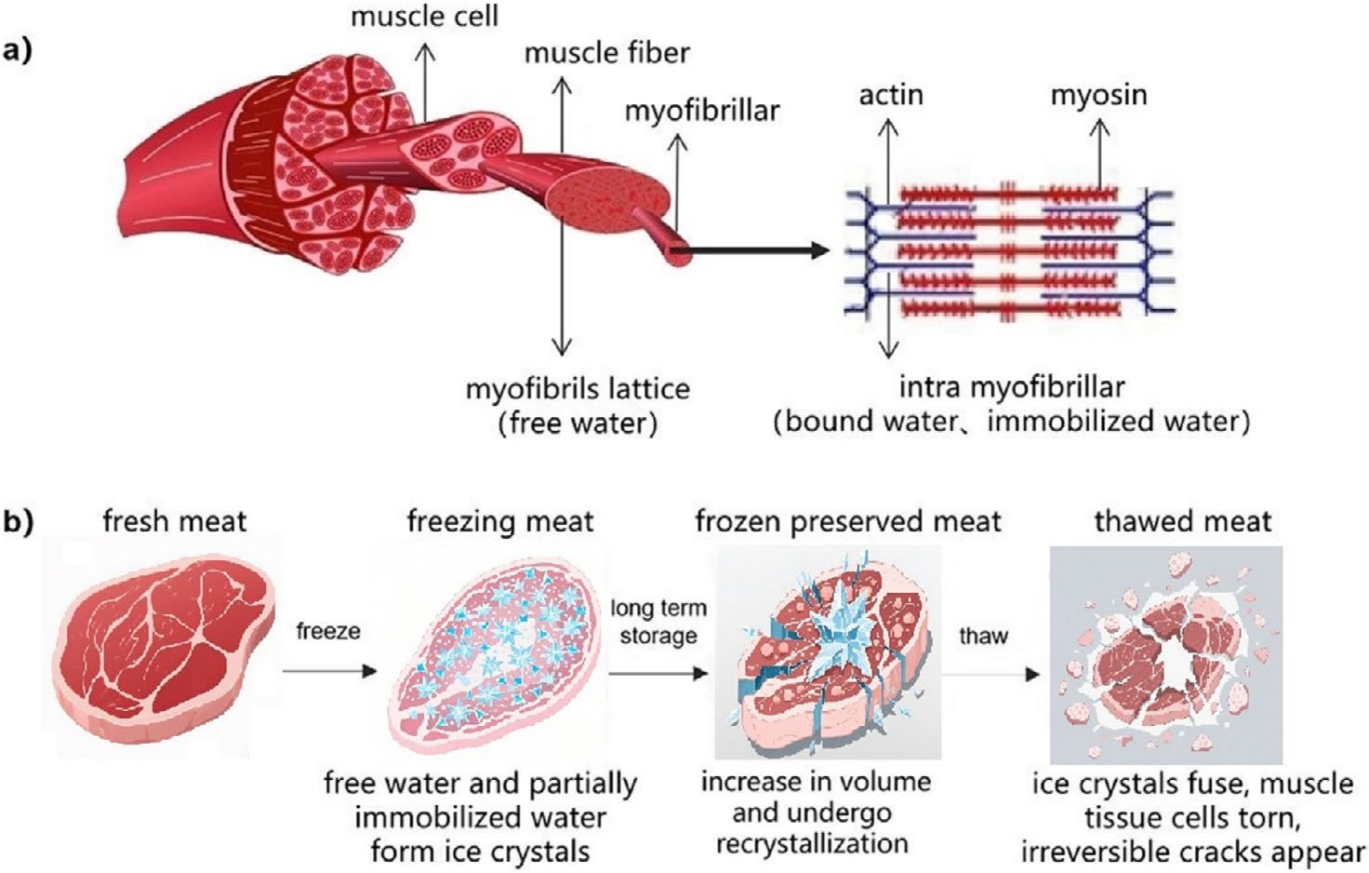
Schematic diagram of water distribution status in muscle and the water–ice–water transition during freeze–thaw processes (Pan et al. 2023). (a) Schematic diagram of water distribution in muscle; (b) Schematic diagram of water–ice–water changes in muscle cells during freezing and thawing.

Fresh meat contains approximately 18% protein and over 75% water. The water is mainly distributed in the spaces between muscle bundles, muscle fibers, endomysium, myofibrils, and myofilaments (Figure 1). Depending on the degree of binding with protein, the water can be classified into free water, immobilized water, and bound water. In unfrozen muscle tissue, the cell fluid is tightly bound to intracellular muscle proteins, maintaining the integrity of the muscle cell or membrane (Du et al. 2022).

Freezing process involves complex phenomena at macro and micro levels, such as nucleation and crystal growth (Shao et al. 2018; Zhang 2019). The rate and interaction of these two stages determine the freezing process and the characteristics of the product. During the critical temperature decline from −1°C to −5°C, a sequential phase transition occurs where free water and immobilized water in the outer muscle layers progressively crystallize while bound water remains molecularly stable (Jia et al. 2022). This thermodynamically significant range, known as the maximum ice crystal formation zone, accounts for the majority of ice formation in muscular tissue. The size distribution and spatial arrangement of ice crystals are governed by multiple processing parameters including freezing rate, temperature profile, and temporal duration (Zhang et al. 2017; Cai et al. 2020). Rapid freezing produces small and uniform ice crystals, which cause less damage to the meat quality (Li, Zhong, et al. 2020). Slow freezing produces large and uneven ice crystals, which cause more damage to the meat quality (Sun et al. 2020). The formed ice crystals undergo dynamic size variations and spatial redistribution within the muscle fiber matrix, continuously modifying their size and dynamic position (Li, Zhao, et al. 2020; Yang et al. 2020). According to Planck's equation, freezing rates are influenced by the thickness of the material, the temperature of freezing medium, and heat transfer coefficients. Direct contact between meat substrates and cryogenic liquids significantly enhances thermal exchange efficiency, thereby accelerating freezing rates (Yang et al. 2020). This complex phase transition process necessitates a multidisciplinary approach that integrates thermodynamic principles, coupled heat–mass transfer mechanisms, and crystallization kinetics, complemented by advanced microscopic visualization and computational modeling (Nikoo and Benjakul 2015). Fractal dimension analysis has emerged as a powerful quantitative tool for characterizing the irregular, complex morphological features of ice crystals (Luan et al. 2018). The water distribution and status in muscle tissues during freezing and thawing are commonly analyzed using techniques such as low‐field nuclear magnetic resonance (LF‐NMR), which distinguishes different water statuses based on T_2_ relaxation time (Zhu et al. 2023), and microscopy to visualize ice crystal morphology (Liu et al. 2025). However, the composition of meat and the formation of ice crystals are highly complex. A comprehensive understanding of these processes requires integrating thermodynamics, mass and heat transfer principles, and crystallization kinetics, along with microscopic imaging and modeling (Liu et al. 2025; Nikoo and Benjakul 2015). Additionally, fractal dimension analysis, an important tool in image processing, can be used to quantitatively analyze the irregular, complex, and random shapes of ice crystals that share similar characteristics (Luan et al. 2018).

Temperature fluctuation during freezing, storage, and thawing leads to the growth and recrystallization of ice crystals in frozen meat, which means that the smaller ice crystals dissolve and adhere to the larger ones, increasing the volume of ice crystals and causing more damage to the tissue structure (Zhu et al. 2019). The complex tissue matrix formed by meat encapsulates most of the water, impeding the transfer of heat and moisture (Li, Zhu, and Sun 2018). During freeze–thaw cycles, ice crystals repeatedly reorganize, causing cell membrane damage. As temperatures rise, ice crystals within cells melt and diffuse into intercellular spaces due to vapor pressure differences. Conversely, when temperatures drop, water molecules form nuclei and aggregate into larger ice crystals, reducing surface energy and creating bulkier crystalline structures (Lin et al. 2022). The greater the temperature fluctuation, the more severe the recrystallization and growth, leading to further damage to the muscle tissue structure (Luan et al. 2018; Zhu et al. 2019). After thawing, cracks appeared in intracellular and extracellular myofibers. The reabsorption of water makes the myofibrils swell again, but it cannot compensate for the damage caused by ice crystals, and the quality and appearance of meat cannot return to the original state (Pan et al. 2020). The increase in porosity leads to the leakage of cell contents and the loss of intercellular water. Additionally, freezing causes the denaturation of myofibrillar protein. As a result, the water holding capacity is reduced, the muscle tissue cannot fully recover during thawing, and the unabsorbed water is lost as drip, affecting the water distribution in the muscle after thawing. Additionally, some nutrients and flavor compounds also flow out with water, resulting in decreased water retention and quality of frozen meat (Wang, Liang, et al. 2020; Zhang et al. 2022).

During thawing, the temperature of muscle tissue is influenced by two factors: ambient temperature and the water phase transition. The ambient temperature determines the rate of thermal exchange between the muscle and the environment, while the water phase transition affects the heat distribution and water status inside the muscle (Cheng, Wang, et al. 2019). When the core temperature of the muscle is below −5°C, the ambient temperature is significantly lower than the muscle temperature, driving rapid heat transfer and causing the muscle temperature to rise quickly. As the core temperature of the muscle reaches −5°C, the water in the muscle begins to undergo a solid–liquid phase transition, changing from ice to water. This phase transition requires the absorption of heat, which slows down the heat transfer and makes the muscle temperature increase more slowly (Zhu et al. 2023). Therefore, during thawing, the change of water status in the muscle when it passes through the phase transition zone has an important influence on the quality of the muscle after thawing.

## 2 Effects of Freezing, Frozen Storage and Thawing on Meat Quality

### 2.1 Protein Oxidation

The formation and growth of ice crystals during freezing cause protein denaturation, which alters the conformation of proteins and exposes some of the hydrophobic aliphatic and aromatic amino acid side chains, reducing the number of protein binding sites with water and affecting the water holding capacity of proteins (Tan et al. 2022; Zhang et al. 2017). Oxidation also facilitates the migration of internal water out of the cells under osmotic pressure (Tippala et al. 2021). The nucleation and growth of ice crystals and their morphology during freezing are the fundamental factors leading to cold‐induced degradation of myofibrillar proteins (Qi et al. 2012; Benjakul and Bauer 2010). The water in the muscle forms ice crystals, and the growth of ice crystals disrupts the integrity of the cells (Dang et al. 2021). The crystallization of water molecules around the myofibrils causes mechanical shear, which weakens the hydrogen bonds between the bound water and the protein side chains, resulting in protein dehydration and folding, as well as damage to the structural stability (Jiang et al. 2019; Hu and Xie 2021). During thawing, pro‐oxidant substances such as heme iron and trimethylamine oxide (TMAO) are released due to cellular disruption. These substances, along with reactive oxygen species (ROS) generated by mitochondrial damage and lysosomal enzyme leakage, contribute to oxidative stress. This oxidative environment promotes protein oxidation by altering protein structural properties, including surface charge, subunit size/composition, disulfide bond formation/breakage, hydrophilic/hydrophobic balance, and molecular mass distribution (Wu et al. 2016; Chen et al. 2018).

The changes of water status (water‐ice‐water) in the muscle caused by freezing and thawing lead to significant changes in the surrounding environment of proteins, such as temperature, solute concentration, pH, and ice crystal abrasion (Qi et al. 2012; Aroeira et al. 2016; Tan et al. 2021). These different stresses cause changes in protein structure and function, affecting their performance.

Freezing and thawing are common methods of preserving food products, which cause changes in the structure and function of proteins. There are mainly two types of protein denaturation that occur during freezing (Zhang et al. 2017). One is the aggregation and denaturation of proteins due to the pressure and oxidation caused by ice crystals. The other is the conformational rearrangement and aggregation within proteins, leading to the disruption of the polypeptide chains. Among the different types of muscle proteins, the myofibrillar proteins are the most susceptible to freeze denaturation, while sarcoplasmic and matrix proteins are relatively stable (Zhang et al. 2021; Miyaguchi et al. 2007). The natural α‐helix structure of the myofibrillar proteins is altered to a random coil during freezing, exposing hydrophobic residues that cause cold denaturation (Tan et al. 2022). This results in a decrease in the salt solubility and thermal stability of the myofibrillar proteins, and an increase in their surface hydrophobicity and solubility (Du et al. 2021). Thawing can partially reverse the denaturation of the myofibrillar proteins, as some of the lost water is reabsorbed by the muscle cells, and the protein hydration and water‐holding capacity are enhanced with the thawing time (Qian et al. 2019). Improper thawing practices critically degrade meat quality by inducing interconnected physicochemical and structural alterations, including moisture loss, protein oxidation, and muscle tissue damage (Xia et al. 2012). Rapid or uneven thawing ruptures cell membranes, releasing myofibrillar water, while temperature fluctuations exacerbate ice crystal recrystallization, mechanically damaging muscle fibers (Xia et al. 2012). Prolonged thawing exposes meat to oxidative stress, where free radicals destabilize myofibrillar proteins by disrupting their tertiary structures, reducing water‐binding capacity and accelerating lipid oxidation. This cascade diminishes the juiciness and sensory quality of meat (Ham et al. 2020; Sun et al. 2019). Extended thawing periods also activate enzymes that degrade cytoskeletal proteins, weakening muscle integrity. Collectively, these processes impair texture, color stability, and nutritional value. Advanced thawing methods, such as radiofrequency, microwave, and high‐voltage static electric field thawing, aim to balance speed with structural preservation, maintaining protein renaturation potential and overall quality (Zhu et al. 2025; Yang, Wu, et al. 2024). Thus, identifying optimal thawing protocols is vital for enhancing protein recovery and meat product quality.

Repeated cycles denature myofibrillar proteins, reducing water‐holding capacity and increasing oxidation (Qi et al. 2012). Freezing rate and pH shifts during freezing critically influence protein denaturation and lipid oxidation (Zhang et al. 2017). Excessive denaturation from multiple cycles compromises protein digestibility by hindering protease activity, diminishing nutritional value. These studies highlight the necessity of optimizing freezing conditions and minimizing freeze–thaw repetitions to mitigate quality losses (Cheng, Wang, et al. 2019). Controlling both freezing and thawing processes is essential to preserve the structural, sensory, and nutritional integrity of frozen meats.

### 2.2 Lipid Oxidation

Lipid oxidation is a process that involves the reaction of unsaturated fatty acids in meat with oxygen, resulting in the formation of unstable free radicals and peroxides, which is one of the major causes of meat deterioration, along with microbial spoilage (Soyer et al. 2010; Chakanya et al. 2017). Lipid oxidation not only affects the sensory and nutritional attributes of meat, but also poses potential health risks to consumers, as some of the oxidation products are toxic or carcinogenic. These compounds cause changes in the flavor, color, texture, and nutritional value of meat, as well as the production of rancid and off odors. Lipid oxidation also affects the interactions of lipids with other meat components, such as proteins and pigments, forming complex molecules that alter the structure and functionality of fats, reducing their solubility, emulsification, and bioavailability (Leygonie et al. 2012; Chen et al. 2018).

The processes of freezing, frozen storage, and thawing can enhance lipid oxidation in meat by increasing the surface area and oxygen exposure, as well as causing temperature fluctuations that promote free radical generation and propagation (Tatiyaborworntham et al. 2022). During freezing, most of the water in meat forms ice crystals, but some biochemical reactions still occur due to the presence of partially unfrozen water, where lipid oxidation is one of the most important factors affecting muscle quality. During frozen storage, the sublimation of ice crystals on the meat surface creates micro‐pores that facilitate the contact of lipids and air, leading to oxidative rancidity and carbonyl‐ammonia reactions, resulting in rancid flavors (Cheng, Wang, et al. 2019). Repeated freezing and thawing cycles change the size and distribution of ice crystals, damaging the cell membrane and organelles, and releasing some pro‐oxidants, such as heme iron, which are closely related to the degree of lipid oxidation (Lv and Xie 2021). The freezing and thawing process also inactivates some antioxidant enzymes that inhibit lipid oxidation, allowing oxidation to occur (Wang, Zhang, et al. 2020).

One of the effective ways to control lipid oxidation is to freeze meat rapidly at low temperatures, which reduces the formation and growth of ice crystals, and preserves the cellular structure and antioxidant activity of meat (Li, Zhu, and Sun 2018; Yang et al. 2020). Moreover, some studies have found that the rate of freezing affects the level of lipid oxidation, with faster freezing methods resulting in lower lipid oxidation (Mulot et al. 2019). Muela et al. compared three different pre‐freezing methods for lamb meat: air‐blast freezing chamber (−30°C), freezing tunnel (−40°C), and nitrogen chamber (−75°C); the result showed that the lamb meat frozen by the nitrogen chamber had the lowest level of lipid oxidation after thawing (Muela et al. 2012). Thawing can induce lipid oxidation in meat, which is the degradation of unsaturated fatty acids by oxygen. Factors such as high temperature, long duration, repeated cycles, and microbial activity during thawing can accelerate lipid oxidation (Köprüalan Aydın et al. 2023).

Packaging methods play a critical role in preserving the quality, nutritional value, and shelf‐life of frozen meat by mitigating oxidation and microbial degradation. Vacuum packaging effectively minimizes oxygen exposure, significantly reducing lipid oxidation rates and suppressing microbial growth, as demonstrated in recent studies (Siddiqui et al. 2024; Zhang et al. 2023). Similarly, modified atmosphere packaging (MAP) extends preservation efficacy through precise gas composition control, which maintains freshness while slowing both microbial proliferation and oxidative deterioration (Zhou et al. 2010). Emerging approaches incorporating antioxidant‐enhanced coatings or edible films have shown additional promise in improving frozen meat stability and sensory quality during extended storage (Putri et al. 2024; Yang, Chen, et al. 2024). These findings emphasize that when combined with appropriate freezing and thawing schemes, optimal packaging is crucial for minimizing quality loss throughout the entire cold chain. The synergy between oxygen exclusion, atmospheric modification, and active preservation technologies underscores the need for method‐specific packaging strategies to address distinct degradation pathways in frozen meat products.

### 2.3 Water Holding Capacity

Water holding capacity is a crucial index for evaluating meat quality, is often quantified by measuring drip loss during thawing, which is closely related to the flavor, color, texture, coagulability, and juiciness of the final product. During the freezing process, the water in intra‐myofibrils forms ice crystals; the formation and growth of ice crystals will cause mechanical damage to the cell membrane and tissue structure, destroying the structure of the myofibrillar, water migrates from the intra‐myofibrils to the inter‐myofibrils spaces, and the melted water of the ice crystals in extra‐myofibrillar space was hard to be completely reabsorbed by myofibrils, cannot be recombined with protein molecules, resulting in the drip loss after thawing (Cheng, Sørensen, et al. 2019; Li et al. 2019).

Numerous studies have investigated the decreased water retention of frozen meat muscle caused by ice crystal growth and protein denaturation (Jiang et al. 2019; Ali et al. 2015). Uneven diffusion and partial reabsorption of drip loss during the freeze–thaw process led to irregular muscle fibers, causing larger ice crystals to reform when refrozen. Broken muscle fibers increase gaps and bundles between the myofibrils, resulting in more severe microstructural damage to muscle tissue after thawing (Dang et al. 2021). The penetration of ice crystals into cells destroys muscle tissue structure. The hydrolysis of protein by released protease accelerates protein oxidation in meat (Sales et al. 2020). Factors such as lipid oxidation to aldehydes and ketones during frozen storage, ATP decomposition to hypoxanthine, and pH decrease caused by glycogenolysis lead to protein denaturation. Oxidation makes water in myofibrillar fibers more likely to migrate under osmotic pressure (Yang et al. 2020; Turgut et al. 2017). Proteins are denatured, and oxidation makes it easier for water to migrate within myofibrils under osmotic pressure, thus increasing thawing loss.

Optimization freezing and thawing methods are crucial for maintaining the water holding capacity and retention of meat products. Different freezing methods, such as slow freezing, fast freezing, high‐voltage electrostatic, and cryogenic freezing, have varying effects on the ice crystal formation, size, and distribution, which in turn affect water loss and meat quality (Jiang et al. 2025; Jia et al. 2017).

Different thawing methods, such as air thawing, water thawing, and microwave thawing, impact ice crystal melting, redistribution, and reabsorption, affecting the water loss and quality of meat products (Llave et al. 2016; Kim et al. 2011, 2018). Rapid freezing methods, such as cryogenic freezing or high‐voltage electrostatic field freezing, produce smaller and more uniform ice crystals, causing less damage to muscle tissue compared to slow freezing (Jia et al. 2022; Li, Li, et al. 2018). Thawing methods also play a crucial role in determining the final meat quality. Microwave thawing, while fast, can lead to uneven heating and protein denaturation, whereas water thawing or air thawing provides more uniform temperature distribution but may result in higher drip loss (Kim et al. 2011; Llave et al. 2016). Optimizing freezing and thawing techniques based on meat type and intended use is essential for maintaining quality and nutritional value. Therefore, it is of great significance to study the proper freezing and thawing methods for the quality of fresh meat.

## 3 Effects of Freezing, Frozen Storage and Thawing on Meat Nutritional Characteristics

**FIGURE 2:**
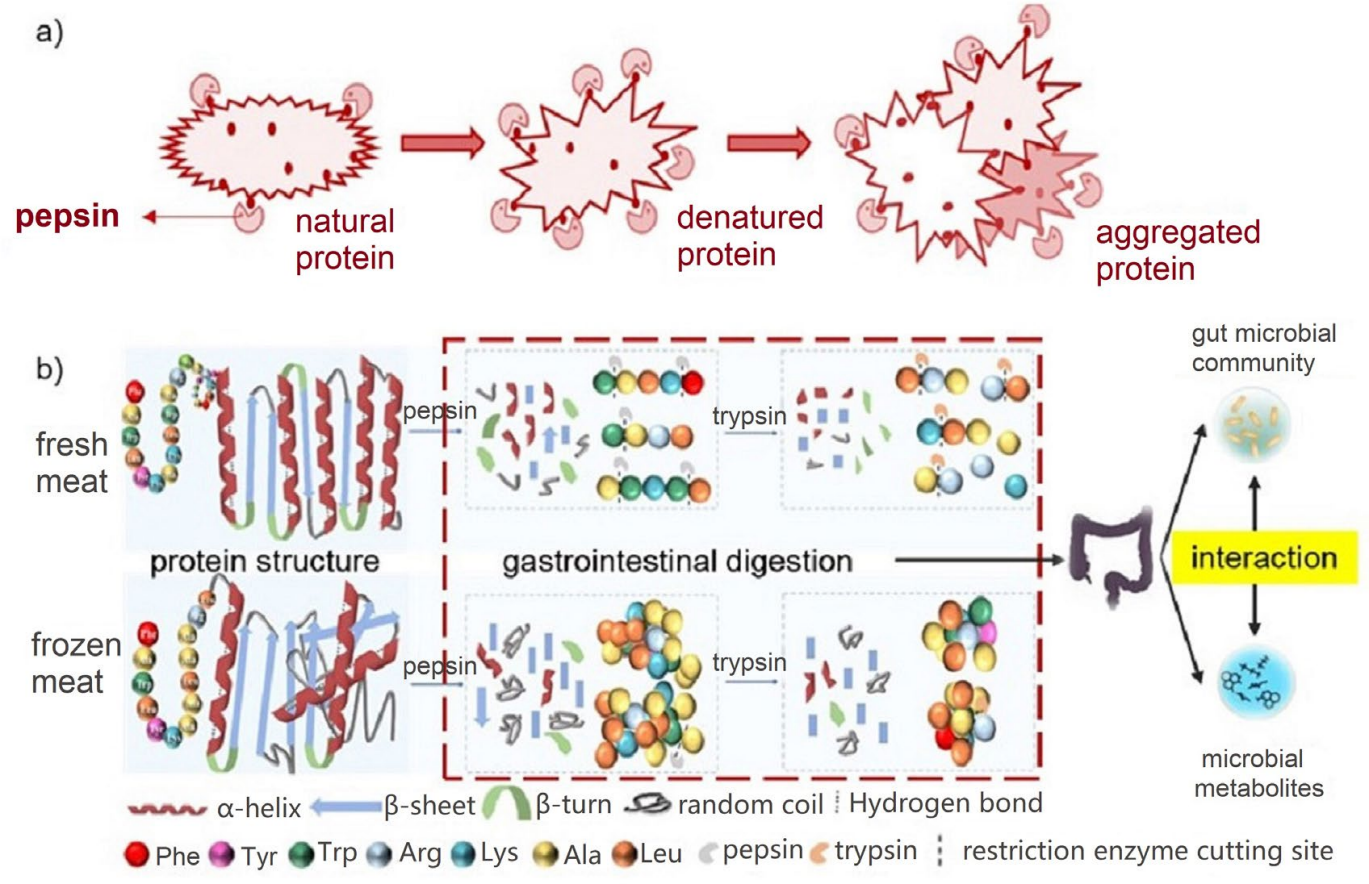
Schematic diagram of protein aggregation affecting the digestion process (Bai et al. 2023). (a) Schematic diagram illustrating differential cleavage site exposure caused by protein aggregation and its effect on pepsin digestion. (b) Schematic diagram of protein simulating gastrointestinal digestion process.

Meat is an important source of dietary protein, and the digestibility of protein determines its bioavailability, which is a fundamental parameter for evaluating the nutritional value of meat. During the freeze–thaw process, protein structures, such as hydrogen bonds, hydrophobic interactions, and disulfide bonds, are destroyed due to cold denaturation, leading to a reduction in α‐helix, an increase in β‐folding formations, a decrease in intrinsic tryptophan fluorescence, and the aggregation of proteins into dense structures that cover enzyme cleavage sites, resulting in reduced digestibility (Du et al. 2021; Bai et al. 2023; Santé‐Lhoutellier et al. 2008). Moderate denaturation and aggregation may expose more cleavage sites, facilitating digestion by gastric enzymes, while excessive denaturation and aggregation can hinder digestion (Figure 2a).

During the freeze–thaw cycle, the structure of the protein is destroyed, the α helix is reduced, the β‐folding is increased, and the intrinsic tryptophan strength is decreased. Pepsin tends to hydrolyze peptide bonds of hydrophobic amino acid residues such as phenylalanine, tryptophan, and tyrosine, while trypsin targets sites of arginine and lysine (Li, Zhao, et al. 2020). Proteins are damaged by oxidation, in which sulfhydryl groups in cysteine and methionine residues are easily oxidized to form desulphated bonds, and tryptophan, tyrosine, and phenylalanine are easily hydroxylated to produce oxidative products such as hydroxytryptophan, dityrosine, and hydroxyphenylalanine (Llave et al. 2016; Kim et al. 2011; Domínguez et al. 2021), thus affecting the recognition of proteases and hindering the digestion of pepsin and trypsin. The effect of freeze–thaw cycle on the digestion characteristics and protein structure characteristics of chicken breast during in vitro digestion was investigated, and it was found that with the increase of freeze–thaw cycle, muscle mastication and shear force increased, while digestibility decreased, and the particle size of digested samples increased (Bai et al. 2023). Compared with fresh muscle samples, the digestibility of gastric digestion and the fifth freeze–thaw cycle samples after gastrointestinal digestion decreased by 25.99% and 11.82%, respectively. Amino acid side chains containing sulfur and aromatic residues are easily oxidized and modified to form covalent cross‐links during the freeze–thaw process, affecting the hydrolysis sensitivity of proteases and reducing protein digestibility (Li, Zhao, et al. 2020). As a result, the freeze–thaw cycle disrupts protein structure and induces protein aggregation, resulting in less digestible material in simulated digestion. Undigested food and endogenous proteins enter the large intestine for use by microorganisms (Figure 2b), thus forming different intestinal microecology and affecting host health (Zhao et al. 2019).

## 4 Conclusion

The impact of freezing, frozen storage, packaging, and thawing on meat quality and nutritional characteristics is a multifaceted process driven by physical and biochemical interactions. Freeze–thaw induces protein denaturation and aggregation, reducing digestibility and bioavailability, while lipid oxidation generates off‐flavors and harmful compounds, degrading sensory and nutritional value. Ice crystal formation, growth, and recrystallization during freezing exacerbate cellular damage, water migration, and functional impairments such as reduced water holding capacity, texture deterioration, color changes, and compromised emulsification. These cumulative effects diminish edible quality, processing performance, and nutritional integrity, with potential health implications for consumers. To address these challenges, optimizing freezing and thawing conditions, employing advanced packaging, incorporating antioxidants, and leveraging novel technologies like high‐pressure processing, ultrasound, microwave, or pulsed electric fields are critical for minimizing quality loss and enhancing nutritional stability.

Future research can focus on the potential mechanisms of meat quality changes during freezing and thawing processes, the application of new freeze–thaw technologies and antioxidants to reduce oxidative damage, the use of advanced packaging materials to extend the shelf life of frozen meat, and cost‐effective innovations, while addressing consumer acceptance and sustainability, to strengthen the frozen meat supply chain and ensure consistent quality and nutritional value from production to consumption.

## Author Contributions

**Yingying Zhu:** conceptualization (equal), resources (equal), writing – original draft (equal), writing – review and editing (equal). **Jiaying Zhu:** writing – review and editing (equal). **Xuebin Shi:** writing – original draft (equal). **Mingdong Fan:** writing – review and editing (equal).

## Conflicts of Interest

The authors declare no conflicts of interest.

## Data Availability

The data that support the findings of this study are available on request from the corresponding author.
